# Supplementary material for: Fecal Transplantation from db/db Mice Treated with Sodium Butyrate Attenuates Ischemic Stroke Injury
Source: Microbiol Spectr. 2021 Oct 6;9(2):e00042-21. doi: 10.1128/Spectrum.00042-21 (PMC8510264; doi:10.1128/Spectrum.00042-21)
Supplement: SUPPLEMENTAL FILE 1 — Supplemental material. Download SPECTRUM00042-21_Supp_1_seq1.pdf, PDF file, 0.4 MB [file spectrum00042-21_supp_1_seq1.pdf]

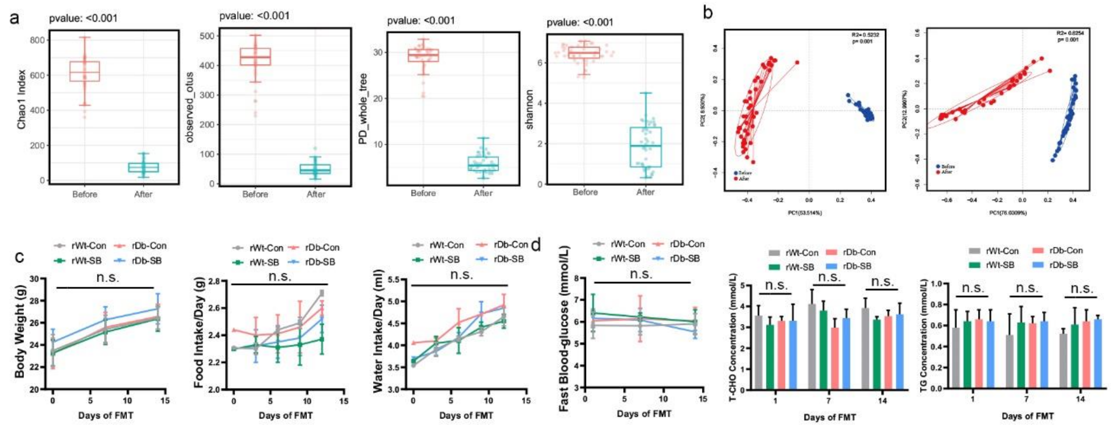

FIG S1 The α- and β-diversity of recipient mice after antibiotic treatment and diabetes-related parameters of recipient mice during FMT. (a) The α-diversity of the four groups measured using the Chao-1, observed OTUs, PD whole tree and Shannon indices (b) PCoA plot of unweighted and weighted UniFrac distances before and after antibiotic treatment. (c) Diabetes-related parameters of the recipient mice during FMT. Data are presented as means ± SD.

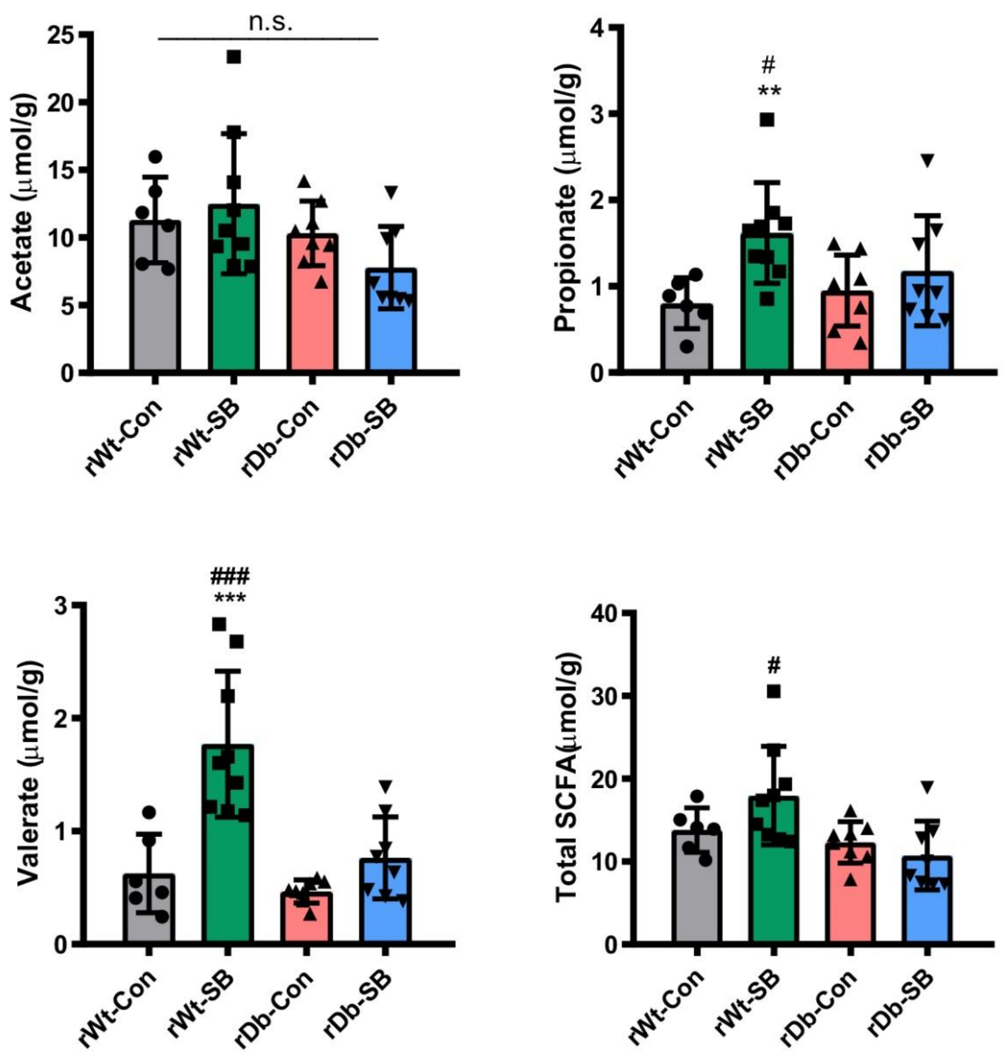

FIG S2 The fecal concentrations of acetate, propionate, valerate and total SCFAs in recipient mice after FMT. N = 6-9 animals per group. Data are presented as means ± SD; *p < 0.05, **p < 0.01, ***p < 0.001, #p < 0.05, ##p < 0.01 and ###p < 0.001. * compared with the rWt-Con group, # compared with the rDb-Con group.

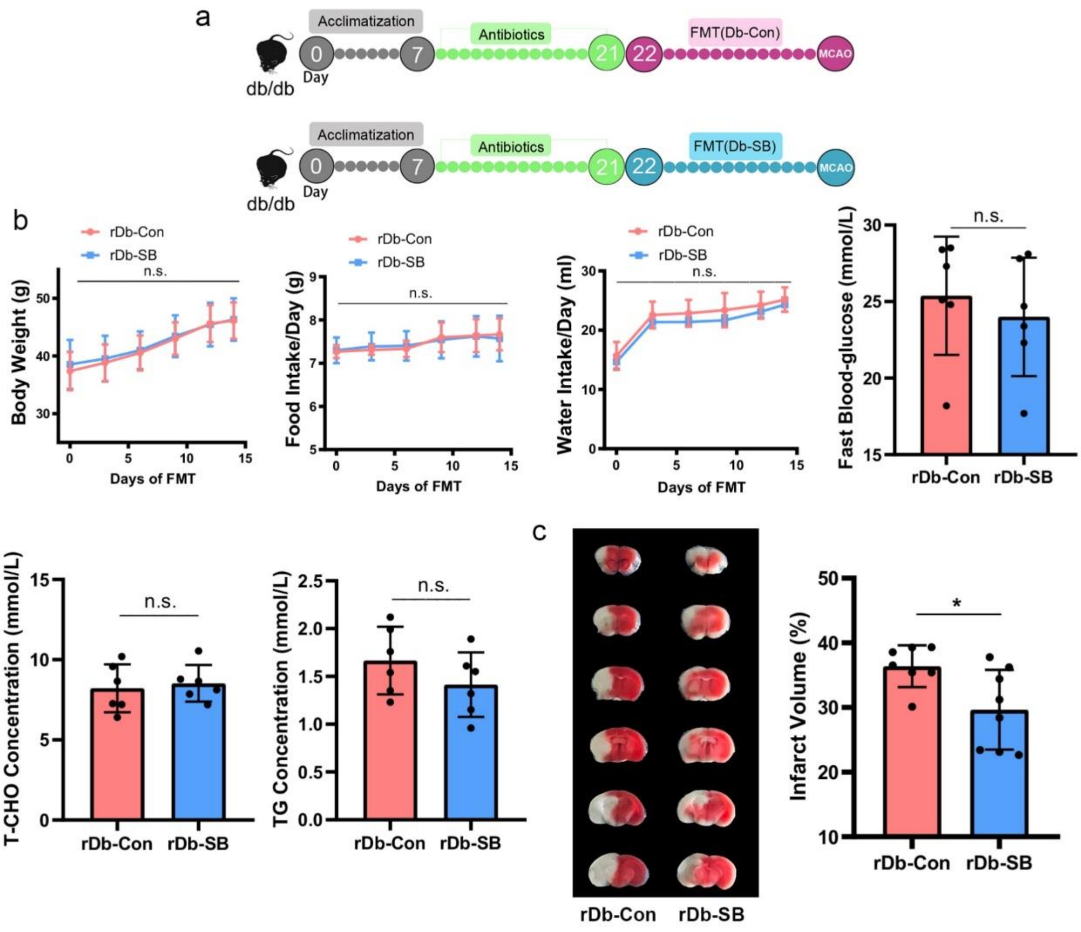

FIG S3 Modulation of the gut microbiota of T2D mice by SB attenuates stroke injury in Db mice independently of a T2D background. (a) Experimental design for Db recipient mice. After acclimatization, the Db recipient mice were administered a cocktail of antibiotics for 14 days and then underwent FMT for an additional 14 days. Diabetes-related parameters were measured during FMT. After FMT, the Db recipient mice were subjected to MCAO for 1h and sacrificed after 24 h of reperfusion. (b) Diabetes-related parameters of the Db recipient mice. (c) Representative image of TTC staining in brain slices and the percentage of cerebral infarct volume in the two groups. N = 6-7 animals per group. Data are presented as means ± SD, *$p < 0.05$.
